# VARIATIONS IN BENEFITS OF INTERGENERATIONAL TUTORING IN THE “NEW NORMAL”

**DOI:** 10.1093/geroni/igac059.2898

**Published:** 2022-12-20

**Authors:** Peter Sun, Nancy Morrow-Howell, Mary Click, Kendra Minch

**Affiliations:** Washington University in St. Louis, Clayton, Missouri, United States; Washington University in St. Louis, St. Louis, Missouri, United States; The Oasis Institute, St. Louis, Missouri, United States; The Oasis Institute, St. Louis, Missouri, United States

## Abstract

This study explored variations in self-perceived benefits of intergenerational tutoring. The study’s sample consisted of 329 older adults who tutored children in-person in the 2021–2022 school year. Due to the COVID-19 pandemic, some of the respondents had experienced a period in which volunteering was remote, virtual, or not possible. Demographic information was collected in a fall pre-test survey, identifying first-time volunteers (first time volunteering in ten years), caregivers (100 or more hours in the last two years helping someone who needed assistance), and male volunteers. Self-perceived benefits of tutoring (physical, emotional, and cognitive health, increased social activities, use time more productively, contribute to the well-being of children, and feel better about myself) were collected in a spring post-test survey. Being a first-time volunteer was significantly associated with improved health (X2 = 4.17, p = 0.041, Cramer’s V = 0.11), even after controlling for baseline self-reported health (p = 0.020). A larger proportion of first-time volunteers (34.2%) reported improvements in at least two areas of health (physical, emotional, and cognitive) due to their involvement in the intergenerational tutoring program, compared to non-first-time volunteers (18.5%). There were no significant differences in perceived benefits for caregivers or males. These findings suggest that targeting non-volunteers for involvement in tutoring programs may maximize health benefits of engagement. Specifically targeting males and caregivers may not be necessary, given that they benefit similarly to other populations.

